# Cancer-associated fibroblasts in pancreatic ductal adenocarcinoma

**DOI:** 10.1038/s41419-022-05351-1

**Published:** 2022-10-25

**Authors:** Tianyi Zhang, Yanxian Ren, Pengfei Yang, Jufang Wang, Heng Zhou

**Affiliations:** 1 University of Chinese Academy of Sciences, Beijing, China; 2 Key Laboratory of Space Radiobiology of Gansu Province & Key Laboratory of Heavy Ion Radiation Biology and Medicine, Institute of Modern Physics, Chinese Academy of Sciences, Lanzhou, China; 3 Department of General Surgery, The First Hospital of Lanzhou University, Lanzhou, China

**Keywords:** Cancer microenvironment, Cancer metabolism

## Abstract

Pancreatic ductal adenocarcinoma (PDAC) is a lethal cancer with a prominent extracellular matrix (ECM) deposition and poor prognosis. High levels of ECM proteins derived from tumour cells reduce the efficacy of conventional cancer treatment paradigms and contribute to tumour progression and metastasis. As abundant tumour-promoting cells in the ECM, cancer-associated fibroblasts (CAFs) are promising targets for novel anti-tumour interventions. Nonetheless, related clinical trials are hampered by the lack of specific markers and elusive differences between CAF subtypes. Here, we review the origins and functional diversity of CAFs and show how they create a tumour-promoting milieu, focusing on the crosstalk between CAFs, tumour cells, and immune cells in the tumour microenvironment. Furthermore, relevant clinical advances and potential therapeutic strategies relating to CAFs are discussed.

## Facts

- Subpopulations of CAFs in PDAC have distinct origins and functions, which can be either tumour-promoting or tumour-suppressing.
- Activated CAFs adapt to and co-evolve with pancreatic cancer cells, influencing PDAC behaviours via paracrine signalling.
- CAFs are orchestrators of the PDAC microenvironment and play a crucial role in helping pancreatic cancer cells thrive in a hostile environment.
- A better understanding of metabolism in CAFs will benefit novel therapeutic paradigms, improving the prognosis of patients with PDAC.

## Open questions

- In the PDAC microenvironment, heterogeneous CAFs serve different functions. How can we tell the difference between distinct subpopulations?
- CAFs are promising targets for anti-tumour interventions. Why do some therapies that target CAFs and ECM result in poorer outcomes?
- CAFs are metabolically linked to tumour cells in PDAC. Is this affecting CAFs’ immunomodulatory capacity? How can we use it to develop novel therapeutic strategies?

## Introduction

Pancreatic cancer, one of the deadliest solid cancers, has remarkably poor prognosis, with a 5-year relative survival rate of only 9% [1]. Pancreatic ductal adenocarcinoma (PDAC), its most common subtype, accounts for approximately 85% of pancreatic malignancies [2]. Despite numerous studies on the disease and the development of new treatment options, early diagnosis and comprehensive treatment for PDAC remain difficult. Most patients are diagnosed at an advanced stage, and the majority of cases are evaluated as unresectable, with only palliative treatment options. Standard chemotherapy regimens for PDAC patients include nab-paclitaxel plus gemcitabine combination therapy or FOLFIRINOX (5-fluorouracil, leucovorin, irinotecan, oxaliplatin), but survival rates have not improved significantly [3]. Furthermore, due to the complex tumour microenvironment (TME), pancreatic cancer exhibits significant resistance to radiotherapy [4]. In contrast, a small subset of PDAC patients with resectable tumours had improved survival after receiving modified FOLFIRINOX (excluding 5-fluorouracil) [5, 6]. However, some patients still have to face a high risk of postoperative recurrence [6]. Hence, it is critical to find an effective treatment as soon as possible.

PDAC typically develops a dense fibrotic stroma with an abundance of extracellular matrix (ECM) due to the inflammation-induced desmoplastic reaction [7]. Cancer-associated fibroblasts (CAFs) are known to be the most important cellular component of the ECM [8]. The stroma is composed of various ECM-related proteins derived from CAFs, including collagen and hyaluronan (HA), and has been linked to intra-tumoural pressure and vasculature [9, 10]. Aside from CAF-mediated TME remodelling, numerous studies have shown that CAFs secrete various paracrine factors that promote tumour invasion, metastasis, and chemoresistance [11]. In the TME, a complex web of signalling connects tumour cells and other cellular components, such as suppressed immune cells like regulatory T cells (T-regs), myeloid-derived suppressor cells (MDSCs), and tumour-associated macrophages (TAMs) [12–14]. CAFs are involved in negative immune regulation, inhibiting the cytotoxic activity of CD8^+^ cells in PDAC, resulting in poor immunotherapy outcomes [15]. Although CAFs have potent tumour-promoting effects, tumour suppressor functions for some CAF subsets also have been reported [16, 17]. It has been demonstrated that CAFs are made up of heterogeneous subtypes that either promote or inhibit tumour growth using single-cell RNA sequencing (scRNA-seq) technology [18, 19]. Although many studies consider CAFs a potential therapeutic target, the lack of highly specific CAF markers makes future research difficult [20]. This review summarises current knowledge about the origins and subtypes of CAFs in PDAC focusing on their functions, such as tumour microenvironment remodelling, metabolic reprogramming, and tumour immunity regulation. Finally, we will go over therapeutic strategies that target CAFs to hasten the transition from bench to bedside.

## Sources and subpopulations of CAFs in PDAC

### Origins of CAFs

**Fig. 1:**
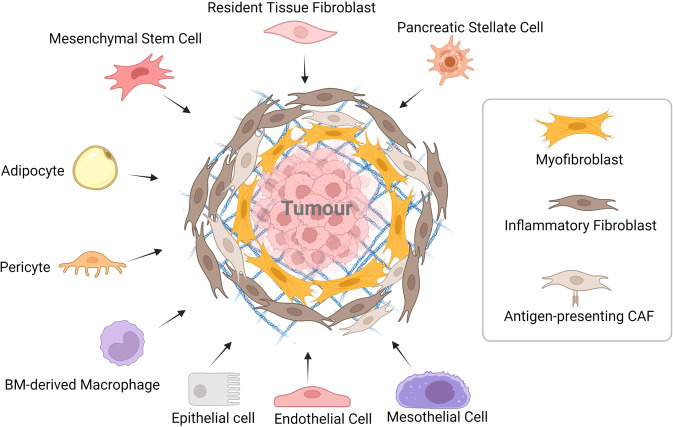
Heterogeneity of CAFs in PDAC. CAFs in PDAC originate from diverse cells. PSCs and other resident tissue fibroblasts can be activated into cancer-associated fibroblasts. Cytokines and chemokines in the TME can cause MSCs, pericytes and adipocytes to differentiate into CAFs and be recruited to the tumour. A subset of BM-derived macrophages concerts into CAF-like cells, promoting pancreatic cancer progression. Transdifferentiation allows epithelial, endothelial, and mesothelial cells to join the CAF population. myCAF, iCAF and apCAF are different phenotypes of CAFs in PDAC that have been discovered. myCAF is spatially located close to the tumour, whereas iCAF is further away from the tumour; apCAF is a smaller subset that promotes immunosuppression in the tumour microenvironment.

It has long been recognised that CAFs represent a heterogeneous population in a variety of malignant tumours [21, 22]. In PDAC, stromal CAFs are often derived from all kinds of cell types, including pancreatic stellate cells (PSCs), tissue-resident fibroblasts, and tumour-infiltrating mesenchymal stem cells (MSCs) [23]. In previous research, PSCs were believed to be the primary progenitor cells of CAFs in PDAC [24, 25]. Approximately 4–7% of typical pancreatic cells are quiescent PSCs, which operate to store vitamin A [26, 27]. Once activated by cytokines, such as transforming growth factor-β (TGF-β), interleukin-6 (IL-6), platelet-derived growth factor (PDGF), and Sonic hedgehog (Shh) [28–31], PSCs lose vitamin A and start expressing α-Smooth Muscle Actin (α-SMA), which is considered a characteristic CAFs marker [32–34]. Unexpectedly, a recent study tracing specific CAF populations in murine models revealed that PSC-derived CAFs represent only a small portion of all CAFs [35]. In addition, it has been shown that adipocytes, pericytes, bone marrow (BM)-derived macrophages, and endothelial cells can differentiate into CAFs and be recruited to the tumour site [36, 37]. Waghray et al. identified cancer-associated MSCs (CA-MSCs) as a specific subpopulation of CAFs [38]. CA-MSCs secrete the granulocytic-macrophage colony-stimulating factor (GM-CSF) exclusively, thereby promoting the progression of PDAC [39, 40]. Moreover, it was discovered that CA-MSCs regulate macrophage polarisation in a tumour-promoting manner. As has been reported recently, BM-derived macrophages are recruited to the pancreas and partially converted into CAF-like cells [41]. CAFs can also be transdifferentiated from non-fibroblastic lineages, such as epithelial and endothelial cells, adipocytes, and pericytes (Fig. 1) [23, 42–44]. Apart from α-SMA, varieties of markers can be utilised to identify activated fibroblasts, including desmin, fibroblast activation protein (FAP), fibroblast-specific protein (FSP1), platelet-derived growth factor receptor (PDGFR), podoplanin, and vimentin [19, 45, 46]. However, these markers are not specific to CAFs in PDAC. Considering that most markers are shared with other non-CAF cell types, further identification of subtype-specific markers is required [18, 36].

### Subpopulations and classification of CAFs

Numerous studies have highlighted the functional heterogeneity of CAFs as a result of the continuous development of technologies [47, 48]. On the basis of their functions, CAFs can be divided into two broad groups: tumour-promoting and tumour-suppressing CAFs [18]. Öhlund et al. made a groundbreaking discovery in 2017, they asserted that CAFs in PDAC exhibit either a myofibroblast (myCAF) or an inflammatory (iCAF) phenotype [49]. myCAFs are located closely to tumour cells, express a high level of α-SMA, and are stimulated by TGF-β. Nevertheless, iCAFs are not only located at a considerable distance from malignant cells, but they also express extremely low levels of α-SMA, elevate production of inflammatory cytokines such as IL-6 and leukaemia inhibitory factor (LIF) [50]. Moreover, the two CAF subsets can transition from one phenotype to the other, indicating that the distinctions between CAF subtypes may be fluid and context-dependent [51]. In colorectal cancer, it was also discovered that CAFs with different phenotypes can switch, indicating the plasticity of CAFs subsets [52]. Multiple preclinical studies have demonstrated that depleting α-SMA^+^ CAFs to reduce fibrotic stroma increases the aggressiveness of tumours [53]. Inhibiting the IL-1/JAK signalling pathway increased the myCAF/iCAF ratio, resulting in a better prognosis, suggesting that a greater understanding of CAFs heterogeneity could contribute to the development of new therapeutic interventions [50]. In addition, antigen-presenting CAF (apCAF), a novel CAF isoform, was identified in human and mouse pancreatic tumours [54]. apCAFs are capable of activating CD4^+^ T cells through the expression of MHC class II and CD74 invariant chain. However, apCAFs do not express classic costimulatory molecules, including CD80, CD86, and CD40, indicating that apCAFs cannot function as expert antigen-presenting cells. Recent research has revealed that apCAFs originate from mesothelial cells and play a role in tumour immunosuppression by inducing the formation of T-regs [55, 56]. Nevertheless, analysis of CAFs in non-small cell lung cancer revealed that apCAFs derived from ATII cells actively promoted immune function. This suggests that cells originating from distinct lineages may have different functions and warrants further investigation [57]. Chen et al. have identified a novel subpopulation of CAFs (complement-secreting CAF, csCAF) adjacent to tumour cells in the stroma of early-stage PDAC. csCAFs express components of the complement system and may regulate the immune and inflammatory response within the tumour [58]. Subtypes of different CAFs, particularly tumour-restraining CAFs, and their specific marker proteins are poorly understood. Hutton and colleagues discovered that the expression of CD105 distinguishes two functionally pancreatic fibroblast lineages in murine and human healthy tissues and tumours [59]. In vivo, CD105^+^ pancreatic fibroblasts promote tumour growth, whereas CD105^-^ fibroblasts suppress tumour growth. It is hypothesised that CD105^+^ and CD105^−^ CAFs derive from distinct spatially resolved precursor fibroblasts; consequently, the contribution of different CAF lineages to PDAC is also a topic worthy of further investigation. In order to develop rational stroma-targeted therapies in the future, it is thus critical to investigate the representative markers of these heterogeneous subpopulations of CAFs (Table 1) [60–62].

**Table 1:** Representative markers of different subtypes of CAFs in PDAC.

CAF markers	CAF subtype	Ref.
Membrane proteins		
FAP	iCAF	[19, 47, 52]
PDGFR (CD147)	myCAF	[51]
MHC Class II	apCAF	[51]
Intracellular proteins		
α-SMA^high^	myCAF	[56]
Desmin	NA	[19, 47]
Vimentin	NA	[19, 47]
Secreted proteins		
IL-6	iCAF	[52]
IL-11	iCAF	[52]
LIF	iCAF	[52]
CXCL12	iCAF	[51]
TGF-β1	myCAF	[51]
Col 1	myCAF	[52]
Complement system components	scCAF	[59]

## Functions of CAFs

**Fig. 2:**
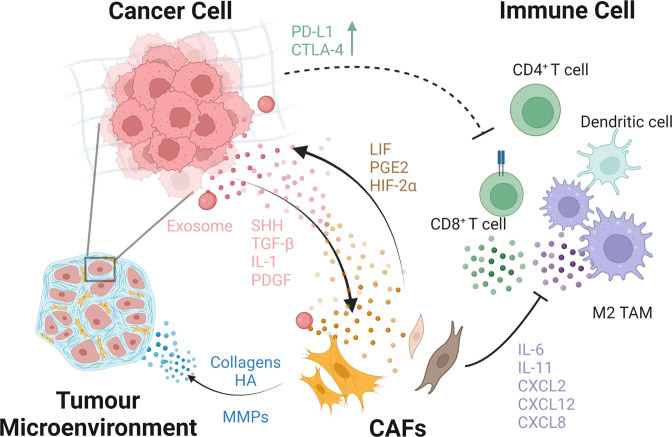
The crosstalk between CAFs, cancer cells and immune cells. CAFs secrete ECM components and contribute to the fibrotic tumour microenvironment. Cancer cell-derived cytokines such as SHH, TGF-β, IL-1, PDGF, and HIF-1 are important in CAF activation, and activated CAFs promote cancer cell proliferation by secreting pro-tumourigenic factors. Furthermore, exosomes released by PDAC cells can aid in the recruitment and activation of CAFs. Pancreatic CAFs contribute to the formation of the inhibitory immune microenvironment by secreting factors such as IL-6, CXCL2, CXCL12, and CXCL8. CAFs are not only responsible for the recruitment and regulation of immunosuppressive cells, but also prevent CD8^+^ T cells from performing anti-tumour functions by upregulating immune checkpoint markers.

Previous research has found that the TME is primarily composed of abundant stroma and diverse cells, thereby forming a physical and metabolic barrier that may impede conventional therapeutic interventions and result in suboptimal therapeutic outcomes [8, 63]. CAFs are the primary stromal cells and the major producers and regulators of ECM components, including collagens, hyaluronan, and proteoglycans [62]. The ECM provides the structure required to support angiogenesis and the associated nutrient supply necessary to support organ function or tumour growth, as has been extensively discussed in previous reviews. [45, 64–66] Additionally, activated CAFs influence neoplastic behaviours through paracrine signalling [67]. CAFs subsets differ in their expression of regulatory cytokines at various stages of PDAC [49, 68], reflecting the functional diversity of CAFs (Fig. 2). Liu et al. demonstrates that the specific functions of diverse stromal CAFs subsets vary with the progression of tumours, being anti-tumour in the early stages and pro-metastasis in the advanced stages [68]. Overall, defining the biological and functional aspects of CAFs at multiple levels and different stages has significant implications for potential therapeutic options [64].

### Responding to tumour signalling

The reciprocal signalling network between CAFs and tumour cells has been well demonstrated [69, 70]. In the TME, PDAC cells secrete diverse factors that activate various signals in the stromal cells. CAFs, in contrast, express paracrine molecules that promote tumour growth and therapy resistance, including growth factors (CTGF, EGF, and PDGF), chemokines (CXCL1, CXCL5, CXCL7, CXCL8, CXCL12, and CCL12), and cytokines (IL-6, IL-11, and LIF) [28–31]. We focus on SHH and TGF-β signalling as examples to illustrate the complex relationship between CAFs and tumour cells in PDAC.

SHH signalling is important in embryonic development and stem cell regulation. Also, it is overactive in PDAC [71]. Sonic Hedgehog could bind to the receptor on PSCs and induce expression of insulin growth factor 1 (IGF1) and growth arrest-specific 6 (GAS6) [72]. Both signalling regulators are secreted and activate Akt signalling in tumour cells, leading to increased cell proliferation and apoptosis resistance. Overexpression of SHH ligands also contributes to the desmoplastic reaction [30]. In a well-defined murine model of PDAC, SHH-deficient tumours had less stromal contents, but were more aggressive, histologically undifferentiated, and had increased vascularity and cell proliferation [20]. Moreover, the treatment with a pharmacological inhibitor targeting SHH signalling attenuates pancreatic cancer growth in mice. It reduces CD8^+^ T cells and increases T-regs, which is correlated with poor prognosis [73]. These results suggest that the role of the SHH signalling pathway is complex in PDAC. In advanced pancreatic cancer, the TGF-β pathway is abnormally activated [74], causing quiescent fibroblasts to transform into CAFs that express α-SMA and inhibit the cytotoxic activity of T cells [75, 76]. TGF-β can also be secreted by activated CAFs in the stroma [77], promoting tumour cell growth and ECM deposition [78]. In addition, TGF-β is responsible for activating STAT3 and MAPK signalling pathways in PDAC [79]. Several preclinical models of solid tumour, including PDAC, demonstrated that halofuginone inhibits collagen synthesis by inhibiting TGF-β signalling [80]. By reprograming T-regs and stimulating CD8^+^ T cell-mediated anti-tumour activity, TGF-β inhibition in conjunction with gemcitabine effectively inhibited tumour development [81]. Of late, it has been revealed that CAFs provide TGF-β to induce tumour progression and chemoresistance by upregulating ATF4 expression [82]. Different regulatory factors play distinct roles in the signalling network of TME (Table 2). A more comprehensive understanding of the interactions between CAFs and malignant cells is critical for developing novel therapeutic strategies that benefit pancreatic cancer patients [83, 84].

**Table 2:** Representative regulatory factors in TME.

Soluble factors	Primary source	Main function	Ref.
Growth factors			
TGF-β	Pancreatic cancer cells iCAFs	Resident fibroblast activation Immunosuppression	[77, 78]
CTGF, EGF, PDGF	CAFs	Promoting cancer cell proliferation	[100]
IGF1	CAFs	Pancreatic cancer modulation	[101]
Cytokines			
TNF-α	Pancreatic cancer cells	Resident fibroblast activation	[100]
IL-1	Pancreatic cancer cells	Resident fibroblast activation	[52]
IL-11	iCAFs	Immunosuppression	[52]
IL-6	iCAFs	Immunosuppression Tumour invasion and metastasis	[52]
LIF	iCAFs	Immunosuppression	[52]
HIF-1α	Pancreatic cancer cells	Metabolic reprogramming	[100]
HIF-2α	α-SMA^+^CAFs	Immunosuppression	[147]
GM-CSF	CA-MSCs	Tumour invasion and metastasis	[39]
Chemokines			
CXCL2	iCAFs	Immunosuppression Tumour invasion and metastasis	[103]
CXCL12	iCAFs	Immunosuppression Reprogramming TME Tumour invasion and metastasis	[102]
CXCL1, 5, 7, 8	iCAFs	Tumour invasion and metastasis	[103]
CXCL18	iCAFs	Tumour invasion and metastasis	[48]
Other factors			
SHH	Pancreatic cancer cells	CAFs modulation	[34]
PGE2	CA-MSCs	Tumour invasion and metastasis	[25]
PD-L1	Pancreatic cancer cells	Immunosuppression	[96, 97]

### Restricting tumour-infiltrated immune cells

In pancreatic tumours, CAFs inhibit immune cell infiltration by generating a dense fibrotic stroma, as has been extensively discussed, but they can also directly modulate the anti-tumour activity of a variety of immune cells. Dendritic cells (DCs), TAMs, MDSCs, T-regs, and cytotoxic T cells make up the PDAC immune microenvironment [85, 86]. The majority of resident immune cells are educated to be immunosuppressive, making PDAC one of the most immunosuppressive tumours [16]. Pancreatic CAFs secrete chemokines, cytokines, and growth factors to recruit and regulate these immunosuppressive cells [87]. Thus, TAMs, MDSCs, and T-regs have been proven to suppress anti-tumoural responses and enhance tumour growth [88, 89]. Furthermore, the dense stroma prevents CD8^+^ T cells from killing tumour cells, resulting in a poor immunotherapy outcome for PDAC [90]. PSCs generate MDSC-promoting cytokines such as IL-6, VEGF, M-CSF, and chemokines (SDF-1, MCP-1) [91, 92]. These factors promote the differentiation of peripheral blood mononuclear cells into MDSCs, then promote tumour progression by suppressing T-cell proliferation and stimulating cancer cell vascularisation and metastasis [92]. Furthermore, a study has shown that CAF-derived LIF also plays an important role in the differentiation of MDSCs [93]. In addition, some activated fibroblasts express fibroblast activation protein-α (FAPα), which cleaves type I collagen (Col 1) and increases macrophage adhesion [94]. These findings suggest that CAFs promote MDSCs differentiation, leading to an immunosuppressive microenvironment.

Tumour cells can be recognised and killed by infiltrating CD8^+^ T cells and evade immune surveillance by inducing T-cell exhaustion [87]. CAFs contribute to immune escape by releasing suppressive cytokines and chemokines, such as IL-6, IL-1β, CXCL1, CXCL2, and CXCL12, and expressing immune checkpoint ligands [95]. Furthermore, as the primary producers of ECM, activated CAFs promote fibrosis, which compresses intra-tumoural vessels and impedes the infiltration of tumour-reactive immune cells [96]. Immune checkpoint ligands, such as CTL-associated antigen 4 (CTLA-4) and programmed death-ligand 1 (PD-L1), bind to effector T cells and contribute to their dysfunction [97, 98]. Gorchs and colleagues demonstrated that Prostaglandin E2 (PGE2) released by pancreatic CAFs inhibits T-cell proliferation and contributes to the upregulation of immune checkpoint markers such as CTLA-4 and PD-1 on activated T cells, resulting in impaired immune function [99]. Another study found that CXCL12, which FAP^+^ CAFs secrete, inhibits the accumulation of cytotoxic T cells in the vicinity of the tumour and may direct tumour immune evasion in a human PDAC model [100]. CAFs enhance the formation of the immunosuppressive microenvironment in PDAC by regulating immune cell activity. However, it is worth noting that different subtypes of CAFs may play distinct roles in this process. Tumour-promoting inflammatory factors, including IL-6, LIF, and CXCL8, are mainly secreted by iCAFs but not myCAFs [50]. In addition, the deletion of Col 1 in α-SMA^+^ myCAFs leads to CXCL5 upregulation in cancer cells, which is associated with recruitment of MDSCs and suppression of CD8^+^ T cells, suggesting that myCAFs slow tumour progression in PDAC [101]. Therefore, further investigations are required to understand better the functional heterogeneity of CAFs in the TME of pancreatic cancer.

### Reprograming tumour metabolism

**Fig. 3:**
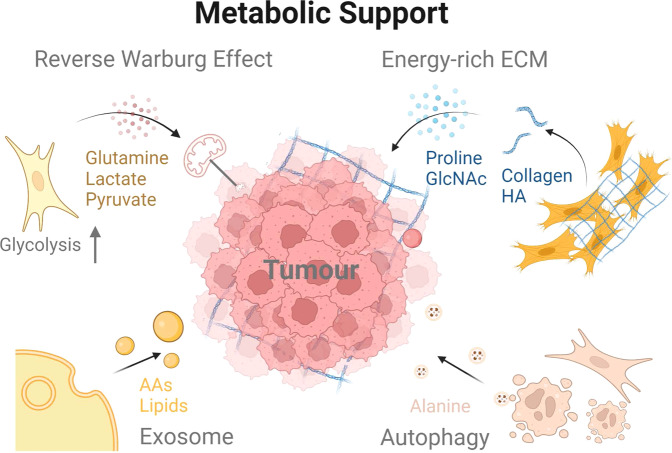
CAFs support metabolic reprogramming of tumour cells. CAFs help malignant cells overcome nutrient deprivation. Under the influence of tumour cells, CAFs perform aerobic glycolysis and provide nutrients to PDAC cells. Moreover, CAFs-derived collagens and HA in the ECM can also be used by tumour cells. Also, CAFs contribute to the metabolism reprogramming of malignant cells. The exosomes released by CAFs can also fuel the metabolism in PDAC cells. Furthermore, CAFs engage in autophagy to generate energy-rich metabolites that serve as alternative carbon sources for mitochondrial metabolism and tumour growth.

In PDAC, the fibrotic stroma limits the availability of nutrients and oxygen [102]. In order to survive, pancreatic cancer cells rewire the metabolic network, switching from oxidative phosphorylation (OXPHOS) to aerobic glycolysis, which is known as the Warburg Effect. Surprisingly, PDAC could hijack nearby CAFs and provide them with energy and nutrients. The ‘Reverse Warburg Effect’ occurs when CAFs are induced to undergo metabolic reprogramming similar to aerobic glycolysis [103]. CAFs secrete energy-rich metabolites such as lactate and pyruvate, which are then taken up by cancer cells and used to fuel OXPHOS, promoting efficient energy production [104, 105]. By interacting with CAFs and other ECM components in the TME, neoplastic cells represent an intricate reprogramming of metabolism [106]. Moreover, CAFs also stimulate glycolytic metabolism via paracrine hepatocyte growth factor (HGF) [107]. Furthermore, through autophagy, CAFs can provide alanine as an alternative carbon source to maintain tumour metabolism and growth [108]. As previously demonstrated, alanine may compete with glucose and glutamine to support OXPHOS and thus nonessential amino acid and lipid biosynthesis in PDAC [109]. Beyond the direct supply of metabolites, CAFs also nourish tumours by producing nutrient-rich ECM. For example, extracellular collagen can be taken up by malignant cells and serve as a source of proline [110]. Recently, Kim et al. found that hyaluronic acid in the ECM can also serve as a nutrient fuel for PDAC metabolism [111]. In addition, by tracking carbon-13-labeled metabolites, Zhao et al. found that CAF-derived exosomes can be taken up by tumour cells in a macropinocytosis-like manner and provide carbon sources such as amino acids and lipids [112]. Collectively, CAFs play a crucial role in helping tumours thrive in a hostile environment (Fig. 3).

## Clinical trials related to CAFs

**Fig. 4:**
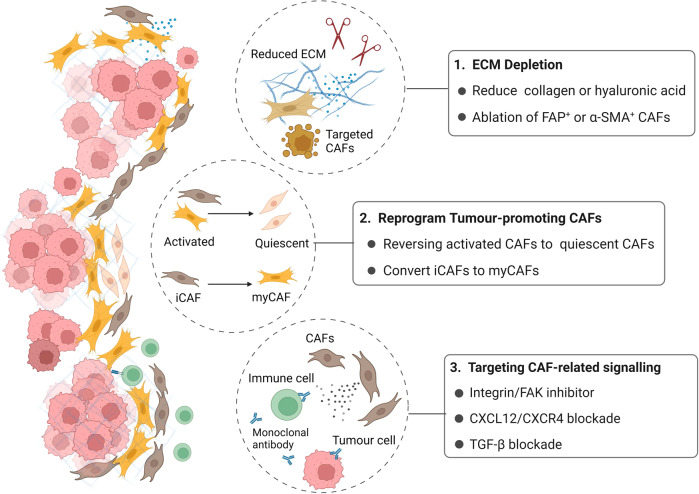
Current treatments related to CAFs. (1) ECM and CAF depletion. Degrade key components of PDAC fibrosis, such as collagen and hyaluronic acid, or ablate specific CAFs subpopulations alone to prevent PDAC desmoplasia. (2) Deactivate or reprogram tumour-promoting CAFs into normalised fibroblasts in order to improve the tumour microenvironment. In some trials, iCAFs are converted to myCAFs to curb tumour progression. (3) Target key cytokines and chemokines and block related signalling in the crosstalk between CAFs, tumour cells, and immune cells, such as FAK signalling, the CXCL12-CXCR4 axis, and TGF-β signalling.

Lumakras (sotorasib), a novel KRAS G12C inhibitor, is being tested in clinical trials and has shown promising results in patients with advanced pancreatic cancer [113]. It is estimated that almost 90% of pancreatic cancer patients have KRAS mutations. However, KRAS G12C accounts for only 1–2% of these mutations, implying that it will benefit only a small percentage of patients with PDAC [114]. As previously stated, CAFs have many tumour-promoting functions in the PDAC tumour microenvironment, including inhibiting drug delivery, metabolic reprogramming, and immunosuppression, making them a promising target for cancer intervention. While our understanding of CAFs is still developing, several preclinical studies and clinical trials have been published. However, given the origin and function heterogeneity of CAFs, developing clinical interventions targeting CAFs still faces numerous obstacles and challenges (Fig. 4).

### Targeting ECM depletion

Since the massive deposition of ECM forms a physical barrier that inhibits chemotherapeutic drug delivery and increases radiotherapy resistance, it is hypothesised that targeting ECM depletion will improve cancer treatment [115]. Among the various components of the ECM, HA and collagens have received the most attention [16]. In preclinical models, however, the absence of Col 1 results in the upregulation of CXCL5 in tumour cells and the recruitment of MDSCs [116]. Losartan was used to target HA, leading to decreased ECM deposition in an orthotopic tumour, which improved patient survival [117]. According to another antifibrotic therapy, the combination of pegvorhyaluronidase-α (PEGPH20) and gemcitabine inhibited tumour desmoplastic reactions and improved overall survival in KPC (*Pdx1-Cre;lox-stop-lox-Kras*^*G12D/+*^*;lox-stop-lox-Trp53*^*R172H/+*^) mice [118]. However, Ramanathan evaluated the activity of PEGPH20 with modified FOLFIRINOX (mFOLFIRINOX) in patients with metastatic pancreatic cancer and found that this combination heightened toxicity and resulted in a shorter treatment duration when compared to mFOLFIRINOX alone [119]. A randomised phase III clinical trial evaluated the efficacy and safety of PEGPH20 with nab-paclitaxel/gemcitabine (AG) in patients with metastatic pancreatic cancer. The combination did not improve OS or PFS [120]; it seems insufficient to target the ECM alone because of poor clinical outcomes, and the dynamic crosstalk between tumour and stromal cells should be considered. In a preclinical research, FAP-specific chimeric antigen receptor T (CAR-T) cells were designed to deplete FAP^+^ CAFs, showing anti-tumour function without significant toxicity [121]. Nevertheless, FAP^+^ cells originating from normal tissues and organs also exhibit highly similar transcriptomic profiles, suggesting that this therapy might lead to the killing of normal cells [122]. Moreover, the inhibition of lysyl oxidase (LOX) by a monoclonal antibody simtuzumab (GS-6624) is also utilised to curb collagen cross-linking and target the protumourigenic stroma [123]. However, simtuzumab in combination with gemcitabine did not achieve significant efficacy in the treatment of pancreatic cancer in adults in a phase II clinical trial [124]. Overall, matrix-targeted agents improve patient outcomes more complex than physical ablation of specific ECM components, so matrix-targeted therapeutic strategies still need further investigation [125].

### Normalisation of activated CAFs

Some researchers are more interested in reprogramming activated CAFs into a dormant state than in CAFs ablation [53]. Since vitamin A deficiency is linked to quiescent fibroblasts activation, restoring retinol levels in PSCs with the all-trans retinoic acid (ATRA) may reverse the state of activated CAFs [126]. Froeling et al. found that ARTA induces quiescence of stromal fibroblasts, with reduced expansion and increased apoptosis of PDAC cells in murine models. When ATRA is combined with gemcitabine, tumour proliferation and invasion decrease, while apoptosis increases compared to the agent alone [127]. Furthermore, calcipotriol, a vitamin analogue, is administered with gemcitabine, resulting in induced stromal remodelling, increased intra-tumoural gemcitabine accumulation, decreased tumour volume, and a 57% increase in survival in KPC mice compared to chemotherapy alone [128]. However, it has been reported that calcipotriol can also up-regulate PD-L1 on cancer cells thereby impairing the anti-tumour function of cytotoxic T cells [129]. Furthermore, some potential mechanisms that aid in the normalisation of CAFs are being investigated. Minnelide is a triptolide analogue with potent bioactivities against a variety of cancers. Dauer et al. discovered TGF-β signalling deregulation in CAFs after Minnelide treatment, resulting in a significant transition from an activated to a quiescent state [130]. Moreover, Lipoxin A4 (LXA4), an endogenous bioactive lipid, inhibits the differentiation of PSCs into CAF-like myofibroblasts and the associated tumour-promoting effects [131]. Thus, efforts to normalise tumour-promoting CAFs or reverse their activated state may open up new avenues for developing novel anti-tumour therapies. In contrast to the fact that PSCs give rise to only a minor subpopulation of CAFs in human PDAC, the majority of related studies have been based on the misconception that PSCs are the major precursors of CAFs [35]. It reminds frontline researchers to focus more on the heterogeneity and diversity of CAFs in primary human tumours. In addition, converting iCAF to myCAF appears to be a promising strategy for ameliorating the immunosuppressive microenvironment. The formation of iCAFs is dominated by IL-1/Jak-Stat signalling, and a Phase I clinical trial of anakinra (IL-1R antagonist) in combination with chemotherapy is currently underway [70, 132].

### Targeting CAF-related signalling

Prior data have highlighted the vital roles of CAF-related signalling pathways in the various stages of pancreatic cancer progression [113, 133]. Integrins have been investigated as pharmaceutical targets for reducing ECM. They are extensively expressed by malignant and stromal cells at focal adhesion. It was reported that inhibiting integrin could significantly slow tumour progression [134]. Monoclonal antibodies that targets integrin, such as Volociximab, has shown therapeutic efficacy in clinical trials to treat pancreatic cancer patients [135]. Furthermore, Jiang et al. discovered that the focal adhesion kinase (FAK) inhibitor VS-4718 may reduce ECM remodelling, while increasing sensitivity to chemotherapy and immunotherapy [136]. Feig and colleagues discovered that FAP^+^ CAFs secreted CXCL12, leading to PDAC immunosuppression [100]. Garg et al. discovered that CXCL12 inhibited cytotoxic T cell infiltration. Blocking CXCL12’s effect on PDAC cells may improve anti-tumour immunity [137]. AMD3100 is an inhibitor of CXCR4 (receptor of CXCL12), and it is reported to promote CD8^+^ T cells infiltration in combination with PD-L1 blockade [100]. Furthermore, TGF-β has emerged as a promising target for the treatment of pancreatic cancer [138]. However, in previous preclinical study, TGF-β blockade increased tumour cell proliferation and accelerated both early and later disease stages [139]. Galunisertib was the first oral TGF-β receptor inhibitor and improved prognosis in advanced PDAC patients in combination with gemcitabine or durvalumab [140, 141]. Notably, it was discovered that TGF-β receptor 2 blockade reduced IL-6 from CAFs, resulting in a reduction of STAT3 activation in cancer cells and improve the anti-tumour immune response [142]. Lan et al. designed a bifunctional protein called M7824, which inhibits tumourigenesis by blocking both PD-L1 and TGF-β signalling. In mouse models, it suppressed tumour growth and metastasis more effectively than treatment with either an anti-PD-L1 antibody or TGF-β trap alone [143]. More research should be done to explore if inhibiting TGF-β signalling in combination with immunotherapy or chemotherapy can improve the prognosis of PDAC, and any side effects from the combination also need to be avoided. In addition, Grauel et al. found that neutralization of TGF-β in vivo led to a dramatic disruption in myCAF activity while boosting the formation of interferon-licensed CAF subsets [144]. It appears that targeting activating signals also contributes to ECM remodelling and is beneficial for enhancing antitumour immunity [145, 146]. However, a better understanding of the role of the ECM in antitumour immunity is required before reliable immunotherapy can be established [147]. Recently, it is found that specific targeting of CAFs-derived HIF-2α can also inhibit cancer cell proliferation and alleviate tumour immunosuppression, providing a new therapeutic target for PDAC [148]. The current therapies involving CAF-targeting agents in pancreatic cancer are summarised in Table 3.

**Table 3:** Clinical trials targeting/relating the CAF in PDAC.

Drug/Target	Mechanism/Strategy	Clinical trial identifies
Losartan	Collagen and HA degradation	NCT03563248, NCT01821729
Simtuzumab	Lysyl oxidase inhibitor	NCT01472198
PEGPH20	Hyaluronic acid degradation	NCT03634332, NCT02241187, NCT01839487, NCT02715804
FAP-CAR T cells	CAF depletion	NCT03932565, NCT01722149
Vismodegib	CAF depletion	NCT01537107, NCT01088815
ATRA	CAF remodelling	NCT03307148, NCT03878524
Minnelide	CAF remodelling	NCT03117920
Anakinra	CAF remodelling	NCT02550327
Paricalcitol	CAF remodelling	NCT04617067, NCT03519308, NCT04524702
High-dose Vitamin D	CAF remodelling	NCT03472833
VS-4718	FAK inhibitor	NCT02651727
Defactinib	FAK inhibitor	NCT03727880
Volociximab	Integrin blockade	NCT00401570
Vismodegib	Hedgehog signalling blockade	NCT01195415, NCT01088815
Sonidegib	Hedgehog signalling blockade	NCT02358161, NCT01485744
Olaptesed pegol (NOX-A12)	CXCL12 inhibitor	NCT03168139
USL311	CXCR4 inhibitor	NCT02765165
Plerixafor (AMD3100)	CXCR4 antagonist	NCT03277209, NCT03277209, NCT02179970, NCT04177810
Motixafortide (BL-8040)	CXCR4 antagonist	NCT02907099
NIS793	TGF-β signalling blockade	NCT05417386, NCT04390763, NCT04935359
Galunisertib	TGF-β Receptor I Kinase Inhibitor	NCT02734160, NCT02154646, NCT01373164
M7824	Bifunctional anti-PD-L1/TGF-β trap	NCT04327986, NCT03451773

## Conclusions and perspectives

CAFs are promising treatment targets because they are the most dynamic and complex components in the pancreatic stroma. On the contrary, CAFs are very heterogeneous, which necessitates further identification and characterisation. Patients with pancreatic cancer may benefit from more specific and personalised therapies if we better understand the diversity of CAFs. CAFs contribute to several features of PDAC, including the deposition of ECM, metabolic support for malignant cells, and immunosuppression. Francescone et al. characterised the functions of Netrin G1 (NetG1) on CAFs in PDAC [149]. NetG1^+^ CAFs not only contribute to immunosuppressive TME, but also allow tumour cells to overcome nutrient deprivation by providing glutamine and glutamate. Specific blockade of NetG1 with a monoclonal antibody inhibits tumour growth and alleviate immunosuppression in mouse models, which also suggests a novel potential target. Furthermore, Wang et al. discovered a new subtype of CAFs with enhanced metabolic activity (meCAF) in PDAC with low desmoplasia, which are characterised as undergoing highly active glycolysis and metabolically coupled with adjacent cancer cells [150]. In addition, PDAC patients with an abundance of meCAFs responded dramatically better to immunotherapy, though more direct evidence is required to further confirm their immunomodulatory function. Francescone et al. demonstrated that inhibiting metabolic-related proteins in CAFs altered their immunosuppressive capacity, linking cell metabolism and immunomodulatory function [149]. As the metabolic link between different subsets of CAFs and tumour cells and immune cells remains elusive, most of the related studies are still in the preclinical stage and there are no reliable clinical translational research results. The role of metabolism-targeted therapy in CAFs should also be emphasised in future research, including the effects on the tumour cells and the modulation of the immune microenvironment. In conclusion, we believe a better understanding of metabolism in CAFs will benefit novel therapeutic paradigms, improving the prognosis of patients with PDAC.

## Supplementary information

Reproducibility checklist

## Data Availability

Data openly available in a public repository (https://clinicaltrials.gov/).
